# Bilateral central serous retinal detachment in a patient with nephrotic syndrome: a case report

**DOI:** 10.1186/s13256-023-04236-x

**Published:** 2023-11-24

**Authors:** Reza Sadeghi, Amirreza Pashapouryeganeh, Morteza Karimi, Elias Khalili Pour, Masoud Mirghorbani, Hamid Riazi-Esfahani

**Affiliations:** Eye Research Center, Farabi Eye Hospital, Tehran University of Medical Sciences, Farabi Hospital, Qazvin Square, Tehran, 1336616351 Iran

**Keywords:** Central serous retinal detachment, Nephrotic syndrome, Systemic lupus erythematosus, Hypoalbuminemia

## Abstract

**Background:**

The aim of this report is to highlight the importance of considering nephrotic syndrome as a potential underlying cause of bilateral central serous retinal detachment in a patient with systemic lupus erythematosus and to underscore the significance of a comprehensive systemic workup in these patients.

**Case presentation:**

A 19-year-old Iranian female patient with history of systemic lupus erythematosus presented with progressive vision loss and bilateral macular elevation. Ophthalmic examination revealed periorbital edema, chemosis, and subretinal fluid at the macula of both eyes. Optical coherence tomography confirmed the existence of subretinal fluid and serous detachment located at the macula of both eyes. On fluorescein angiography, there were no signs of subretinal leakage such as smoke stack sign or expansile dot in late phases. Laboratory tests detected hypoalbuminemia and significant proteinuria, leading to the diagnosis of nephrotic syndrome. Treatment with prednisolone and albumin infusion resulted in improved visual acuity and resolution of subretinal fluid.

**Conclusion:**

Nephrotic syndrome can be a rare underlying cause of bilateral central serous retinal detachment, and its association with systemic lupus erythematosus should be considered. Hypoalbuminemia in nephrotic syndrome alters fluid dynamics in the retina, contributing to bilateral central serous retinal detachment. Early recognition and management of nephrotic syndrome are essential for vision recovery and preventing long-term complications.

## Background

One of the most frequent causes of retinal detachment is serous retinal detachment (RD), which is a result of a variety of ocular and systemic etiologies, including intraocular tumors, posterior scleritis, central serous chorioretinopathy (CSCR), Vogt–Koyanagi–Harada disease, and systemic lupus erythematosus (SLE) [1, 2]. However, hypoalbuminemia is a rare cause of serous RD, and to date, only a few such cases have been reported. Hypoalbuminemia is commonly associated with disorders such as nephrotic syndrome and protein-losing enteropathy (PLE) [3]. We report a case of bilateral central serous RD accompanied by moderate chemosis, which was caused by underlying nephrotic syndrome due to systemic lupus erythematosus (SLE).

## Case presentation

**Fig. 1:**
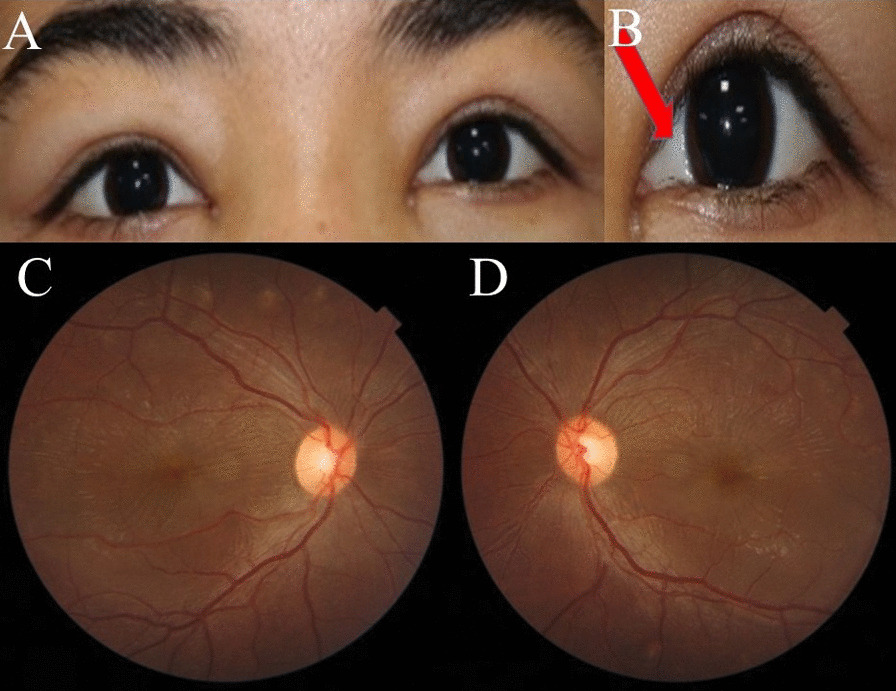
**A**, **B** Slit lamp photographs. Note the moderate chemosis prominent at nasal conjunctiva (arrow) and periorbital edema. **C**, **D** Retinal elevation was observed in both eyes, with macular pigmentary changes more prominent around the vascular arcades

**Fig. 2:**
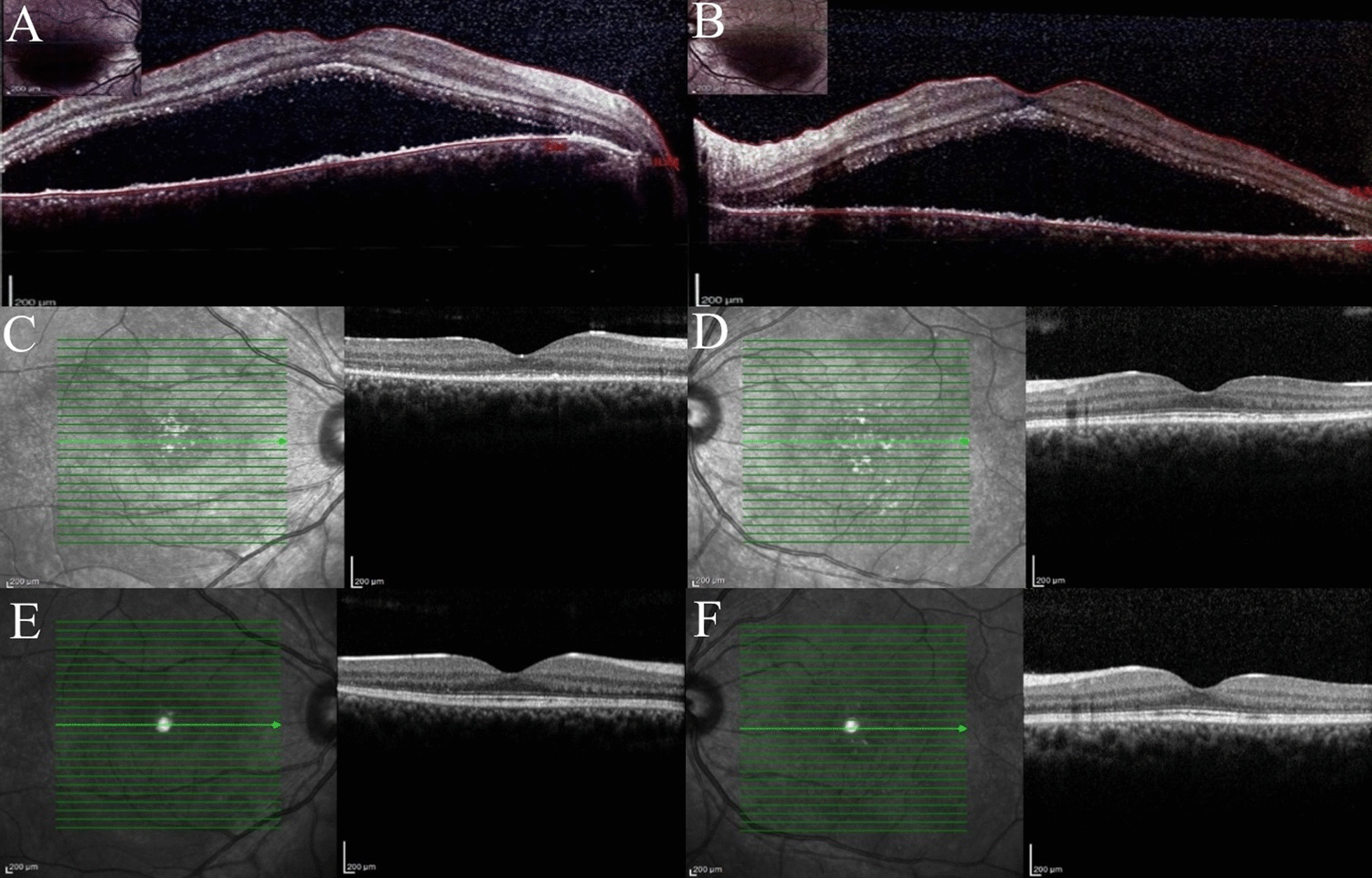
Macular OCT demonstrates fluid accumulation under the retina of both eyes (**A**, **B**). Macular OCT imaging revealed a significant reduction in subretinal fluid in both eyes after 1 month of treatment (**C**, **D**), with restoration of the outer retinal layers observed after 3 months (**E**, **F**)

**Fig. 3:**
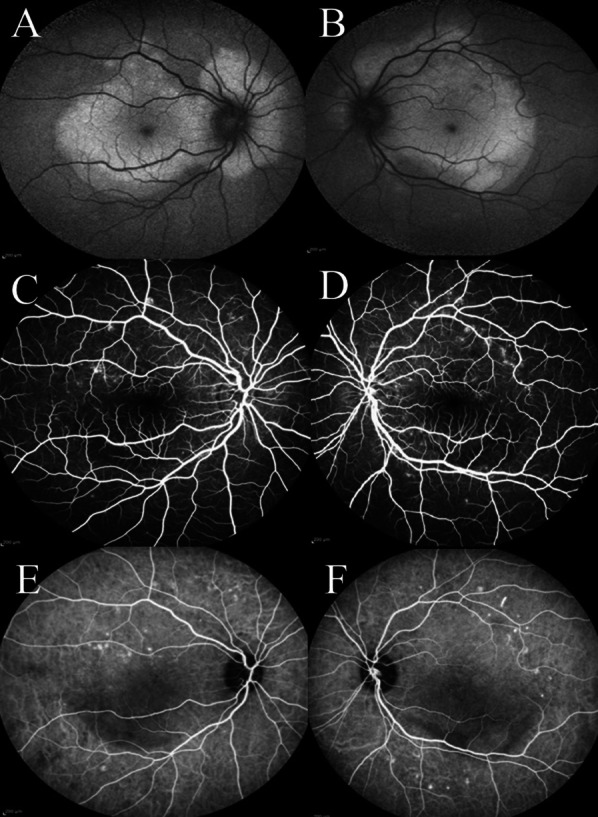
**A**, **B** Fundus autofluorescence imaging demonstrated the presence of hyper-autofluorescent regions in the macula and the surrounding area of the optic nerve head, which is consistent with the presence of subretinal fluid. **C**, **D** On fluorescein angiography, hypofluorescent areas were seen in both eyes because of accumulation of subretinal fluid and the resultant blockage, also staining regions compatible with macular pigmentary changes without any signs of vascular leakage (hyper fluorescence) such as smoke stack sign or expansile dot in late phases (not shown here). **E**, **F** On indocyanine green (ICG), hypercyanescence areas around the vascular arcades show staining compatible with macular pigmentary changes

A 19-year-old Iranian female patient was referred to the Farabi Eye Hospital Retina Clinic with progressive vision loss that had worsened a week before the presentation. Her best visual acuity (BCVA) was 20/50 in the right eye and 20/100 in the left eye. In her medical history, the patient has been diagnosed with systemic lupus erythematosus (SLE) 3 years previously, which had been controlled by 16 mg of dexamethasone daily. Notably, she had no history of pregnancy. In terms of social and environmental factors, the patient had no known exposure to environmental toxins or other factors that could contribute to her current ocular symptoms. Her family history was unremarkable for any significant ocular or autoimmune disorders, and there was no known familial history of SLE. The patient was not employed at the time of presentation. Upon presentation, the patient's vital signs were as follows: pulse rate of 62 beats per minute, blood pressure measured at 120/75 mmHg, and body temperature recorded at 36.5 °C. During ophthalmic examination, periorbital edema and moderate chemosis were observed in both eyes, with the chemosis being more prominent in the nasal conjunctiva (Fig. 1A, B). Fundoscopic examination revealed bilateral macular elevation in both eyes (Fig. 1C, D). The results of other ocular assessments, including the anterior segment, lens, and intraocular pressure (IOP), exhibited no abnormalities. Optical coherence tomography (OCT), fundus autofluorescence (FAF), fluorescein angiography (FA), and indocyanine green angiography (ICG) confirmed the existence of subretinal fluid and serous detachment located at the macula of both eyes (Figs. 2, 3). On FA, there was no signs of subretinal leakage such as smoke stack sign or expansile dot in late phases (Fig. 3).

A comprehensive systemic assessment was carried out, encompassing a detailed analysis of the patient’s biochemistry profile. The results demonstrated metabolic stability, as evidenced by urea (29 mg/dl; reference range 17–55) and creatinine (1.16 mg/dl; reference range 0.67–1.71) levels within the expected range, affirming unimpaired renal function. Lipid parameters, including total cholesterol (152 mg/dl; desirable rang: < 200) and triglycerides (115 mg/dl; desirable range < 150), fell within recommended healthy thresholds. Hepatic function markers, such as ALT (SGPT) at 16 U/l and AST (SGOT) at 15 U/l, displayed no notable aberrations. Electrolyte levels, comprising calcium (9.7 mg/dl), sodium (141 mmol/l), and potassium (4.0 mmol/l), were meticulously maintained. Hematology assessments revealed normalcy, with a white blood cell count (WBC) of 4.5 × 10^3^/µl, specific neutrophil (2.5 × 10^3^/µl) and lymphocyte (1.5 × 10^3^/µl) counts, alongside red blood cell parameters, notably RBC count (4.88 million/µl), hemoglobin (Hb) level (14.8 g/dl), hematocrit (Hct) level (44.2%), and mean corpuscular volume (MCV) (90.6 Fl), all well aligned with established norms. Platelet count comfortably resided within the anticipated range at 210 × 10^3^/µl. Immunoassay results pertaining to thyroid function, including T4 (6.51 µg/dl), T3 (101.6 ng/dl), and TSH (1.005 µIU/ml), consistently adhered to their respective reference ranges. Furthermore, laboratory tests identified a decreased serum albumin level (2.4 g/dl; normal range, 3.5–5.5), while urine analysis unveiled significant proteinuria at 4 + level, with a 24-h urine sample registering 1324 mg of proteins, markedly exceeding the conventional range, which typically remains below 150 mg. As hypertensive chorioretinopathy was a differential diagnosis, the blood pressure was measured and found to be within the normal range. Also, there was no evidence of hypertension in the the patient’s previous documents. The patient was referred to a nephrologist for further evaluation. A kidney biopsy and all laboratory tests regarding kidney function were performed to determine the patient’s diagnosis. The patient was ultimately diagnosed with nephrotic syndrome attributed to systemic lupus erythematosus (SLE). The patient was managed with oral administration of 50 mg/day of prednisolone and an intravenous infusion of albumin at a dosage of 1mg/kg to reverse hypoproteinemia. During re-examinations conducted 1 and 3 months later, the patient’s visual acuity had improved to 20/32 and 20/25 in right and left eyes, respectively. Additionally, both eyes showed a notable decrease in subretinal fluid upon examination with OCT after 1 month, and the outer retinal layers was restored after 3 months (Fig. 2C–F). At the 6-month follow-up, there were no complaints of vision loss, and all ophthalmic examinations were unremarkable.

## Discussion and conclusion

This case presents a distinctive instance of serous retinal detachment (RD) associated with hypoalbuminemia in the context of nephrotic syndrome. Unlike more common causes of serous RD, this 19-year-old female patient with known SLE history displayed bilateral central serous RD and chemosis, yet without hypertensive crisis or subretinal leakage on fluorescein angiography (FA) images. The rarity of this presentation underscores the importance of considering nephrotic syndrome in the differential diagnosis of serous RD, particularly when accompanied by these unique clinical features.

Eye involvement associated with systemic lupus erythematosus (SLE) encompasses various conditions such as eyelid involvement and orbital inflammation in the form of periocular lesions, keratoconjunctivitis sicca, or secondary Sjögren’s syndrome, episcleritis, and scleritis [3] Additionally, patients may experience unilateral or bilateral retinopathy, retinal detachment, secondary angle-closure glaucoma, and ischemic optic neuropathy [3]. Serous retinal detachment in systemic lupus erythematosus (SLE) is infrequently reported. However, Dalmarco Ghem *et al.* documented a case where serous retinal detachment appeared as an initial sign of lupus choroidopathy [1].

Instances of systemic hypoalbuminemia causing serous detachment are limited, and the underlying causes vary between reported cases. In a publication by Mafi *et al.* [4], a case of protein-losing enteropathy (PLE) was documented, where the patient exhibited bilateral serous retinal detachment (RD) and presented with visual acuity reduction. After administering an oral dose of prednisolone at 30 mg per day and adherence to a high-protein and low-fat diet, the serous retinal detachments (RDs) gradually regressed over a 2-month follow-up period. As a result, the patient’s visual acuity showed significant improvement. Herrington Wong *et al.* reported two cases of nephrotic syndrome with bilateral serous RD, who was treated successfully with prednisolone in one case and cyclosporine A in another case. In both cases, the serous RD was resolved with marked improvement of vision in 2 months after treatment [5]. De Benedetto *et al.* reported a 24-year-old female who was referred with generalized edema secondary to nephrotic syndrome and had bilateral blurring of vision with serous RD that entirely resolved with diuretic therapy in just 1 week [6]. A case with a similar presentation due to nephrotic syndrome secondary to minimal change disease has been reported by Henriques *et al.* Although the manifestations were refractory to systemic diuretics, 2 months after hemodialysis visual acuity improved to 20/25, and near-normal restoration of retinal anatomy was achieved, with concurrent remission of proteinuria [7].

The outward flow of fluid from the vitreous across the retina and retinal pigment epithelium (RPE) is one of the main contributing mechanisms in maintaining the adhesion of the neurosensory retina to the RPE [4]. It has been suggested that the oncotic pressure of the choroid, along with the active ion-fluid transport pump of the RPE cells and the intraocular pressure, plays a significant role in maintaining flowability [8, 9]. Serum albumin is the primary determinant of the oncotic pressure within the circulating system, including the choroidal vessels [4]. In cases such as PLE and nephrotic syndrome, a decline in serum albumin levels reduces oncotic pressure in the choroidal vessels. This reduction, in turn, triggers transudation and fluid accumulation in the subretinal space.

The distinction between this cause from CSCR was essential since the approach to treatment is completely different. In this case, no obvious leakage was observed in fluorescein angiography (FA) images, which is a characteristic sign of CSCR [4]. This is in contrast to cases of CSCR, where the pathophysiology is mainly caused by enhanced hydrostatic pressure in choroid and deficient retinal pigment epithelium (RPE) pumping the fluid from the subretinal space into the choroid [4]. Instead, there was blockage without any pinpoint leakage or other hyperfluorescence symptoms, such as the smoke stack sign or expansile dot, in the locations where subretinal fluid had accumulated. On the basis of our findings, we postulate that the reduced oncotic pressure hypothesis is a plausible explanation for the subretinal fluid accumulation observed in this patient. Furthermore, the absence of hyperfluorescence on fluorescein angiography suggests that alternative pathophysiological mechanisms can be ruled out [4]. The marked improvement with oral steroids supported the diagnosis of neurosensory RD secondary to hypoalbuminemia. If the underlying condition of serous retinal detachment were due to CSCR, treatment with systemic steroids would worsen the eye condition and the vision. Conversely, serous detachment due to hypoalbuminemia would respond very well to systemic corticosteroids [5].

Notably, nephrotic syndrome may present as a prevalent manifestation of systemic lupus erythematosus (SLE) [10]. Physicians should include systemic lupus erythematosus (SLE) in their list of differential diagnoses for patients who present with serous retinal detachment (RD) since it is a condition that may occur with SLE concomitantly. The observation of bilateral central serous detachment in patients without any hypertensive crisis should lead to suspicion of hypoalbuminemia, primarily if it is accompanied by additional signs such as chemosis and the absence of leakage on fluorescein angiography (FA) images. Timely identification of systemic lupus erythematosus (SLE) and nephrotic syndrome is crucial to restoring visual function and initiating early treatment for both ocular and systemic symptoms associated with the condition.

## Data Availability

The datasets used in the current study are available upon reasonable request.

## Abbreviations

RD: Retinal detachment
SLE: Systemic lupus erythematosus
CSCR: Central serous chorioretinopathy
PLE: Protein-losing enteropathy
BCVA: Best visual acuity
IOP: Intraocular pressure
OCT: Optical coherence tomography
FAF: Fundus autofluorescence
FA: Fluorescein angiography
ICG: Indocyanine green angiography
RPE: Retinal pigment epithelium
